# Supplementary material for: WordCommentsAnalyzer: A windows software tool for qualitative research
Source: F1000Res. 2018 Sep 4;7:536. Originally published 2018 May 3. [Version 2] doi: 10.12688/f1000research.14819.2 (PMC6137411; doi:10.12688/f1000research.14819.2)
Supplement: Supplementary file 3 [file f1000research-7-17614-s0002.tgz › b2a79496-4ada-47c4-9682-a99ce867836d.zip!/Eight Iranian Health Journals/Journal of Research in Rehabilitation Sciences _51.docx]

© Journal of Research in Rehabilitation Sciences . This work is licensed under CC BY-NC 4.0 (https://creativecommons.org/licenses/by-nc/4.0/).

Volume & Issue: دوره 4، شماره 1: 1387

Title: مقایسه ضخامت قدامی– خلفی عضلات شكم در ورزشکاران حرفه‌ای دو سرعت و استقامت با استفاده از اولتراسونوگرافی

http://jrrs.mui.ac.ir/index.php/jrrs/article/view/23

مقدمه: هدف از این مطالعه، بررسی تفاوت ضخامت قدامی- خلفی عضلات شكم در دو گروه ورزشکاران حرفه‌ای دو سرعت و استقامت بوده است. مواد و روش‌ها: 18 نفر از ورزشکاران حرفه‌ای دونده، شامل 9 نفر سرعتي و 9 نفر استقامتي، در این مطالعه شرکت كردند. در طي يك مطالعه توصيفي- تحليلي، اندازه‌گیری ضخامت عضلات شكم با استفاده از دستگاه اولتراسونوگرافی انجام گردید. داده‌های مربوط با استفاده از پرسش‌نامه تحقیق و دستگاه اولتراسونوگرافی جمع‌آوری شده، اختلاف بین دوگروه با استفاده از آزمون t مستقل مورد تحلیل آماری قرارگرفت. یافته ها: ضخامت قدامی- خلفی کلیه عضلات شكم در دو گروه اختلاف معني‌داری را نشان داد (001/0 P <)؛ به گونه‌ای که عضلات استقامتی در گروه دوندگان استقامت و عضلات حرکت دهنده در گروه دوندگان سرعتي از ضخامت بیشتری نسبت به گروه دیگر برخوردار بود. بحث: نتایج این مطالعه نشان داد که تمرین ورزشی دراز مدت اثر اختصاصي بر روی عضلات پوسچرال و عضلات حرکت دهنده دارد؛ به نحوي که ورزش استقامتي، بیشترين تأثير را روی عضلات استقامتی و ورزش سرعتي، بیشترين تأثير را بر روی عضلات سرعتی می‌گذارد.

Title: بررسی شيوع مشکلات مفصل ساکروايلياک در مبتلايان به کمردرد مراجعه کننده به مراکز پزشکی شهر اصفهان (يک مطالعه مقدماتي)

http://jrrs.mui.ac.ir/index.php/jrrs/article/view/24

مقدمه: کمردرد يكي از دلايل شايع از كارافتادگي است كه از يك سو سبب اختلال در آسايش فرد و از سوي ديگر سبب تحميل هزينه‌ای گزاف بر فرد و جامعه می‌‌شود. يکي از علل کمردرد مشکلات مفصل ساکروايلياک است که به دليل همراه بودن با درد و علايم ناحيه کمري، تشخيص آنها بسيار دشوار است و در اغلب موارد در طي روند ارزيابي و درمان، ناديده گرفته مي‌شود. در تحقيق حاضرسعی شد شيوع اين مشکلات در بيماران مبتلا به کمردرد مراجعه کننده به مراکز پزشکی شهر اصفهان بررسی شود تا به عنوان يک مطالعه پايه از اطلاعات حاصل براي طراحي و اجراي يک مطالعه شيوع سنجي در سطح شهر اصفهان استفاده گردد. مواد و روش‌ها: در این مطالعه، وجود مشکلات مفصل ساکروايلياک در بيماران مبتلا به کمردرد که در ماه‌هاي تير و مرداد 1386 به مراکز پزشکی الزهرا (س)، آیت ا... کاشاني و امين مراجعه کردند، بررسي گرديد. نمونه‌گيري به روش احتمالي ساده انجام و حجم نمونه با توجه به مطالعات اپيدميولوژيک مشابه، 114 نفر در نظر گرفته شد. یافته ها: بيش از 30 درصد مبتلايان به کمردرد همزمان از مشکلات مفصل ساکروایلیاک (SIJ) نیز رنج مي‌بردند. نتيجه‌گيري: درSI  با توجه به شیوع بالای مشکلات مفصل نه تنهادر مبتلایان به کمردرد⁮ توجه و بررسي علايم اين اختلال در بيماران مبتلا به کمردرد بسيار حائز اهميت است، بلکه کوتاهي در اين مسير مي‌تواند از دلايل عمده عدم رضايت این گونه بيماران از درمان‌هاي فيزيوتراپي باشد.

Title: بررسی تأثیر ورزش هوازی بر اختلال افسردگی در زنان بزرگ‌سال– تهران (1387)

http://jrrs.mui.ac.ir/index.php/jrrs/article/view/25

مقدمه: اختلال افسردگی از اختلالات شایع دوران بعد از بلوغ می‌باشد. از رایج‌ترین روش‌های درمانی در این اختلال، دارو درمانی و روان درمانی است. با توجه به عوارض جانبی و نیز وقت‌گیر و گران بودن این روش‌ها، توسعه درمان‌های غیر دارویی در سطوح مختلف این اختلال، ضروری به نظر می‌رسد. هدف از پژوهش حاضر، بررسی تأثیر ورزش هوازی به عنوان نوعی روش درمان در زنان بزرگ‌سال (45-18 سال) با اختلال افسردگی بود. مواد و روش‌ها: در این پژوهش مداخله‌ای و شبه‌تجربی، 82 نفر از زنان بزرگ‌سال توسط پرسش‌نامه افسردگی بک II از میان 150 نفر انتخاب شدند. 41 نفر به عنوان گروه آزمون به مدت 4 هفته و هفته‌ای 3 روز به مدت 1 ساعت در ورزش هوازی شرکت کردند. در این مدت 41 نفر شرکت کننده در گروه شاهد که شامل افراد قرار گرفته در لیست انتظار بودند، در هیچ فعالیت ورزشی شرکت نکردند. بعد از طول مدت مداخله دوباره پرسش‌نامه مربوط از افراد گرفته شد. یافته ها: افراد شرکت کننده در ورزش هوازی در مقایسه با گروه شاهد و نیز نسبت به قبل از انجام ورزش، کاهش معني‌داری را در شدت افسردگی نشان ‌دادند (05/0 P <)؛ به گونه‌ای که با توجه به تست بک II، میزان افسردگی از شدت متوسط به شدت خفیف کاهش یافته بود. در صورتی که در گروه شاهد، شدت افسردگی تغییر نکرده بود. نتیجه‌گیری: 4 هفته ورزش هوازی در کاهش افسردگی در زنان بزرگ‌سال مؤثر بوده، می‌تواند به عنوان یک روش درمانی در کاردرمانی این اختلال مورد استفاده قرار گیرد.

Title: بررسی ارتباط بين پروناسيون پا و زاويه كوادريسپس در افراد مبتلا به سندروم درد مفصل پتلوفمورال با استفاده از کفی طبی عملکردی

http://jrrs.mui.ac.ir/index.php/jrrs/article/view/26

مقدمه: درد قدامي زانو يكي از بیماری‌هاي شايع در بین افراد عادي و ورزشكاران است. یکی از عوامل ايجاد این بیماري‌ها، مشکلات ساختاری اندام تحتانی و از جمله زاويه كوادريسپس است. چنان‌چه مطالعات نشان مي‌دهد در افراد مبتلا به درد قدامي زانو ميزان زاويه كوادريسپس با ميانگين افراد، متفاوت است. به لحاظ تئوري يكي از روش‌هاي كاهش زاويه كوادريسپس، كاهش زاويه پروناسيون مفصل ساب تالار است كه از طريق استفاده از كفي‌هاي عملكردي به دست مي‌آيد؛ این امر يكي از مؤثرترين روش‌هاي درمان افراد مبتلا به درد مفصل پتلوفمورال كه در ضمن داراي پروناسيون بيش از حد مفصل ساب تالار هستند، نيز مي‌باشد. اما تحقيقات تجربي اندكي در تأييد تأثيرات اين ارتزها وجود دارد. مواد و روش‌ها: تعداد 18 نفر بيمار مبتلا به سندروم درد مفصل پتلوفمورال كه داراي پروناسیون بيش از حد پا بودند، با انجام مراحل خاص غربالگري وارد مطالعه شدند. زاويه كوادريسپس و زاويه والگوس پاشنه اين افراد قبل و بعد از استفاده از كفي عملكردي اندازه‌گيري شد. داده‌ها با استفاده از آزمون paired t-test مورد ارزيابي قرار گرفت. يافته‌ها: میانگین زاويه كوادريسپس در هر دو پا، كاهش معني‌داري بعد از استفاده از ارتز عملکردی نشان داد (02/0 = P). همچنین زاویه واگلوس پاشنه پا در هر دو طرف به طور معنی‌داری کاهش یافت (04/0 = P). نتيجه‌گیری: اصلاح پروناسيون پا با ارتزهاي عملكردي مي‌تواند باعث كاهش زاويه كوادريسپس و بهبود علايم بيماري در قدامي زانو شود.

Title: تعيين فركانس سه سازه اول واكه⁯هاي زبان فارسي در دانشجويان 24-18 ساله دانشگاه⁯هاي علوم پزشكي اصفهان و دانشگاه اصفهان

http://jrrs.mui.ac.ir/index.php/jrrs/article/view/27

مقدمه: هدف از اين مطالعه تعيين فركانس سه سازه اول واكه⁯هاي زبان فارسي در دانشجويان 24ـ18 ساله دانشگاه⁯هاي علوم پزشكي و اصفهان و دستیابی به یک ایده⁯ال از فرکانس سازه⁯های واکه⁯ها و نسبت میان آنها در افراد طبیعی بزرگسال مي⁯باشد. مواد و روش⁯ها: اين پژوهش توصيفی به صورت مقطعي روي 60 نمونه (30 نفر پسر و30 نفر دختر) از ميان دانشجويان فارسي زبان دانشگاه⁯هاي علوم پزشكي اصفهان و دانشگاه اصفهان در محدوده سني 24-18 ساله انجام گرفت. ابتدا اطلاعات زمینه⁯ای توسط مصاحبه مستقيم گردآوري و سپس آزمون توليد واکه⁯هاي زبان فارسي از نمونه⁯هاي مورد نظر گرفته شد. سپس سازه اول و دوم و سوم هر 6 واكه به طور جداگانه توسط دستگاه «Speech Studio» و برنامه real analysis نرم⁯افزار Dr. Speech به دست آمد و اعداد مربوط به هر فرد به طور جداگانه در جدول مربوطه ثبت گرديد و در نهايت بر اساس اهداف و سؤالات پژوهش مورد بررسي قرار گرفت. یافته ها: در دانشجويان پسر ميانگين بيشترين مقدار  Fمربوط به واكه /æ/، 767 هرتز و 2Fمربوط به واكه/i/، 2244 هرتز و 3Fمربوط به واكه/i/، 3129 هرتز و كمترين مقدار 1F مربوط به واكه /i/، 255 هرتز و 2Fمربوط به واكه/u/، 734 هرتز و 3Fمربوط به واكه /u/، 2317 هرتز به دست آمد. در دانشجويان دختر ميانگين بيشترين مقدار 1F مربوط به واكه /æ/، 876 هرتز و 2Fمربوط به واكه /i/، 2688 هرتز و 3Fمربوط به واكه /i/، 3339 هرتز و كمترين مقدار 1F مربوط به واكه /u/، 376 هرتز و 2Fمربوط به واكه/u/، 731 هرتز و 3Fمربوط به واكه/u/، 2412 هرتز به دست آمد. بحث: واكه‌هاي /i/ و /u/ بسته و واكه⁯هاي /æ/ و/a/ باز هستند و دو واكه /e/ و /o/ بين اين دو گروه قرار گرفتند. واكه⁯هاي /i/، /e/ و /æ/ پيشين و واكه⁯هاي /a/،/o/ و /u/ پسين هستند. واكه⁯هاي /i/ و /æ/ گسترده و واكه /e/ نيمه‌گسترده می‌باشد. واكه⁯هاي /a/ و /u/ گرد و /o/ نيم‌گرد است.

Title: بررسی تأثیر تمرینات فرانکل بر روی آتاکسی، تعادل، فعاليت‌هاي روزمره و افسردگی بیماران مبتلا به آتاکسی مخچه‌ای ناشی از مولتیپل اسکلروزیس

http://jrrs.mui.ac.ir/index.php/jrrs/article/view/28

مقدمه: مولتیپل اسکلروزیس شایع‌‌ترین بیماری نورولوژی پیشرونده در جوانان بالغ می‌‌باشد. این بیماری باعث تخریب میلین در آکسون‌های عصبی شده و در نتیجه ضایعات عملکردی ایجاد می‌کند. یکی از مناطق مهم درگیری در MS، مخچه می‌باشد. اختلال در این ساختمان منجر به ایجاد آتاکسی، اختلال در تعادل و ... شده و به تبع آن کیفیت زندگی بیمار را کاهش می‌‌دهد. تمرین درمانی، یک روش مؤثر درمانی در کاهش اختلالات عملکردی بیماران MS می‌‌باشد. اين مطالعه تأثير تمريناتي فراوان را در بهبود شرايط بيماران مبتلا به MS نشان مي دهد. مواد و روش‌‌ها: این مطالعه از نوع آینده‌ نگر، شبه‌تجربی و یک سویه ‌کور بوده و به منظور بررسی اثر تمرینات فرانکل بر آتاکسی، تعادل، ADL (Activity of Daily Living) و افسردگی بیماران MS، در سال 86-85 در شهر اصفهان انجام گرفته است. در این مطالعه طی 10 جلسه، به 22 بیمار مبتلا به درگیری مخچه‌‌ ناشی از MS، (با شرایط دموگرافی سن:36/7± 36/33 سال، قد:36/8 ±14/164سانتیمتر، وزن: 95/8±95/61کیلوگرم و مدت زمان ابتلا:92/50±18/62ماه) به صورت یک روز در میان تمرینات فرانکل آموزش داده شد. این تمرینات روزی دو نوبت و هر نوبت، 30 دقیقه توسط بیمار تکرار می‌‌گردید. اطلاعات حاصل از انجام آزمونها، قبل از درمان و بعد از درمان جمع‌آوری گردید. به منظور بررسی آتاکسی از Ataxia Rating Scale، برای تعادل از Berg Balance Scale، برای ADL از Barthel Index و جهت ارزیابی افسردگی از پرسش‌‌نامه افسردگی Beck استفاده شد. جهت تجزیه و تحلیل آماری نتایج به دست آمده از آزمون  tزوج شده موجود در نرم‌‌افزار آماري SPSS استفاده شد. یافته ها: پس از بررسی‌های آماری نتایج زیر حاصل گردید: بین وضعیت (05/0P <)آتاکسی، وضعیت تعادل (05/0P <)، وضعیت ADL (05/0P <) و وضعیت افسردگی (05/0P <) بیماران، قبل و بعد از درمان تفاوت معنی‌‌داری وجود داشت. بحث: نتایج حاصل از این مطالعه نشان داد که انجام تمرینات فرانکل به صورت مداوم، با فرض ثابت ماندن شرایط بیمار، باعث بهبودی در شاخص‌‌های آتاکسی، تعادل، ADL و افسردگی بیماران مبتلا به MS می‌‌شود. با توجه به روند بیماری، در صورت توقف انجام تمرینات، شرایط بیمار به وضعیت اولیه بر خواهد گشت. کلید وا ژه‌‌ها: مولتیپل اسکلروزیس، آتاکسی مخچه‌‌ای، تعادل، ADL، افسردگی و تمرینات فرانکل

Title: روايي و پايايي ترجمه فارسي فرم كلمات و ژست‌ها از فهرست تكامل برقراري ارتباط مك آرتور- بيتز

http://jrrs.mui.ac.ir/index.php/jrrs/article/view/29

مقدمه: هدف از ";فهرست تكامل برقراري ارتباط";، تهیه ابزاری است که اطلاعات معتبری را در زمینه رشد و تکامل زبان از حرکات بیانگر غیر کلامی تا تكامل واژگان كودك در سال‌های اولیه زندگی و سپس تا شروع دستور زبان ارائه دهد. هدف كلي اين طرح، تعيين روايي و پايايي ترجمه فارسي فرم كلمات و ژست‌ها (براي كودكان 8 تا 16 ماهه) از اين فهرست بوده است. مواد و روش‌ها: مطالعه از نوع توصيفي- تحليلي و مقطعي بود. پس از يك پيش آزمون و محاسبه ضريب همبستگي، گويه‌هاي آزمون با يكديگر و نيز مشورت با صاحب‌نظران و والدين، فرم تجربي اوليه تهيه و وارد مطالعه شد. نمونه مشتمل بر 30 كودك 8 تا 16 ماهه طبيعي بدون مشكلات مشخص ژنتيكي و ذهني بود كه با مراجعه به مراكز بهداشت شهر اصفهان به شيوه نمونه‌گيري غيراحتمالي انتخاب شدند. پرسش‌نامه اطلاعات پايه و فرم كلمات و ژستها توسط والدين تكميل گرديد.  نتايج به منظور تعيين روايي صوري و محتوايي با استفاده از محاسبه همبستگي دروني سؤالات آزمون با يكديگر و با خرده مقياس‌ها و براي تعيين ضريب پايايي با معيار آلفاي كرونباخ مورد بررسي قرار گرفت. يافته‌ها: روایی صوری و محتوای آزمون CDI با در نظر گرفتن نقطه نظرات 2 زبان‌شناس و 2 آسیب شناس گفتار و زبان و والدین مشخص گردید. بيشترين ضرايب پايايي محاسبه شده (98/0) براي خرده مقياس درك واژگان و كمترين ضرايب پايايي محاسبه شده (43/0) براي ژست‌هاي كلي بود. بحث: به نظر مي‌رسد زياد بودن تعداد گويه‌هاي مربوط به خرده مقياس درك واژگان و پر بسامد بودن گزارش مادران درباره درك كودكانشان و كمترين ميزان هم‌خواني فرهنگي در خرده مقياس ژست‌ها مي‌تواند دلايلي بر اين يافته‌ها باشد. اما به طور كلي، ضرايب هر دوي اين خرده مقياس‌ها و نيز بيان كلمات با نمونه انگليسي مشابهت داشت. دومين فرم تجربي اين فهرست، آماده‌ انجام مطالعات آينده است.

Title: بررسی تأثیر استفاده از دستگاه "Audiovisual Tactile" بر روی بهبودی حسی پس از ترمیم عصب اولنار

http://jrrs.mui.ac.ir/index.php/jrrs/article/view/30

مقدمه: اين مطالعه با هدف جانشین ساختن حس شنوايی با حس لمس از دست رفته به طور زود هنگام پس از ترميم عصب اولنار در دست، انجام گرفت. این کار از نظر تئوريكي به حفظ نقشه کورتیکال دست در کورتکس سوماتوسنسوری در طی دوره اوليه آوران‌زدايی كمك نمود. مواد و روش‌ها: دستگاه ";Audiovisual Tactile"; بلافاصله به‌دنبال ترميم عصب اولنار به‌منظور بهبودي حساسيت‌ پذيري دست، از طريق حفاظت فعالانه نقشه دست در كورتكس سوماتوسنسوري در طي دوره آوران‌زدايي، مورد استفاده قرار گرفت. این دستگاه به‌طور زود هنگام پس‌از ترميم عصب اولنار در يك مرد 25 ساله استفاده شد. ارزیابی حسی در فواصل منظم، 2، 5 و 7 ماه پس از شروع مداخله انجام شد. یافته ها: نتایج حاکی از بهبودی حس دست در بیماری که درمان‌شده با حس ساختگی، نسبت به بیمار دیگر بود. بحث: این مطالعه نشان داد که زمان شروع تأمین درون‌داد حسی، با تأکید بر یادگیری مجدد حسی، فاکتور بسیار مهمی می‌باشد.

Title: گزارشی از نظام بهداشت و درمان کشور انگلستان

http://jrrs.mui.ac.ir/index.php/jrrs/article/view/31

مقدمه: امروزه نظام بهداشت و درمان، یکی از معیارهای پیشرفت جوامع به شمار می‌رود؛ به گونه‌ای که کشورهای دارای شاخص‌های درمانی استاندارد، جزء کشورهای پیشرفته و برخوردار و ملل فاقد این گونه شاخص‌ها در رده کشورهای عقب‌افتاده و غیر برخوردار قرار می‌گیرند. با وجود پیشرفت‌های قابل ملاحظه‌ای که طی سه دهه اخیر در زمینه‌های مختلف بهداشتی و درمانی در کشور ایران به وقوع پیوسته و جایگاه کشورمان در بین کشورهای رو به توسعه بسیار ارتقا یافته است، هنوز راهی طولانی جهت نیل به استانداردهای بهداشتی و درمانی فرا روی ما قرار دارد. طبق گزارش سازمان بهداشت جهانی، خدمات ملی بهداشت و درمان انگلستان (NHS) یکی از بهترین و کامل‌ترین نظام‌های بهداشتی و درمانی دنیا، به لحاظ حفظ استانداردهای بین‌ المللی و ارتقا سطح سلامت جامعه، به شمار می‌رود. هدف از نگارش این گزارش، معرفی روش‌های تعامل بیماران و مجموعه شاغل در نظام بهداشت و درمان انگلستان می‌باشد. بازگویی نکات برجسته و پرداختن به مسايل حاشیه‌ای این نظام از دیگر اهداف این گزارش مي‌باشد. بحث: این نوشتار ضمن بازگویی نکات قابل توجه در نظام بهداشت و درمان انگلستان، شرایط را برای مقایسه این مجموعه با نظام مشابه آن در کشور ایران فراهم می‌سازد. امید است که این گزارش بتواند نکات مثبت موجود در نظام بهداشت و درمان انگلستان را به درستی منعکس ساخته، راه را برای انطباق این نکات در کشورمان تا حد امکان به وجود آورد.
